# The Question of the Origins of COVID-19 and the Ends of Science

**DOI:** 10.1007/s11673-023-10303-1

**Published:** 2023-09-11

**Authors:** Paul A. Komesaroff, Dominic E. Dwyer

**Affiliations:** 1 Faculty of Medicine, Nursing and Health Sciences, Monash University, Clayton, VIC 3800 Australia; 2 New South Wales Health Pathology—ICPMR Westmead, University of Sydney, Westmead Hospital, Westmead, NSW 2145 Australia

**Keywords:** COVID-19, SARS-CoV-2, Origins hypotheses, Politics

## Abstract

Intense public interest in scientific claims about COVID-19, concerning its origins, modes of spread, evolution, and preventive and therapeutic strategies, has focused attention on the values to which scientists are assumed to be committed and the relationship between science and other public discourses. A much discussed claim, which has stimulated several inquiries and generated far-reaching political and economic consequences, has been that SARS-CoV-2 was deliberately engineered at the Wuhan Institute of Virology and then, either inadvertently or otherwise, released to the public by a laboratory worker. This has been pursued despite a clear refutation, through comprehensive genomic analysis, of the hypothesis that the virus was deliberately engineered and the failure of detailed investigations to identify any evidence in support of a laboratory leak. At the same time a substantial, established body of knowledge about the many factors underlying the emergence of novel zoonotic diseases has been largely ignored—including climate change and other mechanisms of environmental destruction, tourism, patterns of trade, and cultural influences. The existence and conduct of these debates have raised questions about the vulnerability of science to manipulation for political purposes. Scientific discourses are vulnerable because: (i) claims can be made with no more than probabilistic force; (ii) alleged “facts” are always subject to interpretation, which depends on social, ethical, and epistemological assumptions; and (iii) science and scientists are not inherently committed to any single set of values and historically have served diverse, and sometimes perverse, social and political interests. In the face of this complexity, the COVID-19 experience highlights the need for processes of ethical scrutiny of the scientific enterprise and its strategic deployment. To ensure reliability of truth claims and protection from corrupting influences robust ethical discourses are required that are independent of, and at times even contrary to, those of science itself.

## Introduction

One of the most troubling aspects of the global response to the COVID-19 pandemic has been the manner in which it has been used by various regimes to achieve political, social, or economic advantages or to divert attention from their own pandemic responses (Brewster 2021; Cohen 2022a, b, c; Mazzetti, et al. 2020). Commentaries about the origins of SARS-CoV-2, purportedly drawing on scientific arguments, have served as key devices in this process.

While factually discredited and ultimately unsuccessful, these attempts to undermine the probity of science and scientists have nonetheless helped focus attention on both the factors underlying the emergence of pandemics and the nature and role of science in the contemporary world. They have raised ethical questions about how science can be used and, at times, manipulated. The discussion is important because the impact not just of the pandemic itself but also of the way in which it has been handled are likely to have long-lasting consequences. In particular, the divisions and hostility deliberately fomented at a time of unprecedented crisis may ultimately be seen as a major lost opportunity for the establishment of global processes to address deep threats to humanity.

The use and abuse of science by politicians (and, by association, their general public supporters) in these debates may have been egregious but they are not new. Science has always been both a force for human benefit and for destruction and domination. It has always contributed to progressive causes and been used by malign political regimes to reinforce and protect their power. While the story of the role of science in the response to the current pandemic has yet to be told in full, enough is known already for critical lessons to be drawn for the future.

## The Emergence of COVID-19

Some of the bare facts are not in dispute (Maxmen 2021; SAGO 2021; Schnirring 2020). It is widely agreed that COVID-19 was first identified after a number of cases were recognized and traced to exposure at the Huanan wet market in Wuhan on December 9, 2019. Nine cases were identified and a public announcement was made by the Chinese Government on December 30, 2019. The report of the isolation of the causative agent was published on January 7, 2020 and the first full viral sequence three days later. The World Health Organization (WHO) announced a global health emergency on 30, January 2020 and a pandemic was announced on 11, March. During the following months the disease quickly spread around the world, often overwhelming hospitals and causing large numbers of deaths.

Multiple SARS-CoV-2 genomic variants have since emerged, and the disease continues to cause high levels of suffering and death in many countries around the world. While the “official” death toll at present stands at around seven million, it is accepted that the actual number of deaths—including the excess mortality linked directly or indirectly to COVID-19—is at least three times this figure, with the most reliable estimates placing the total number of excess deaths associated with the epidemic in September 2022 at about twenty-five million.

Much attention has been applied to the first few weeks of the pandemic in Wuhan. While the time scale is relatively short—indeed, some have observed that few other countries in the world could have achieved a more rapid or efficient response—it was nonetheless undoubtedly prolonged by local political and cultural factors. Early reports of a new, long awaited epidemic similar to SARS were vigorously, and falsely, denied by officials, losing precious time. A whistleblower, Dr Li Wenliang, who tried heroically to draw attention to the mounting danger, was ignored and then victimized—only later, sadly, to be honoured posthumously for his courage.

Although the SARS-CoV-2 genome had been sequenced in late December, showing unequivocally the novel nature of the infective agent, more days were lost before it was made available to the world—and then (some have argued) only as a result of outside pressure. Although evidence supporting person to person transmission was recognized early, official statements (including from the WHO) continued to question whether this was the case for at least a further three weeks (Harrison and Sachs 2022).

Arguably, if the Chinese administrative processes had been more flexible and less bureaucratic and scientists had been allowed to communicate more openly and directly (within and outside China), the process of responding to the developing crisis in both China and world-wide could have been significantly expedited. Having said this, much that occurred subsequently—including the failed public health response in several countries and the resultant rapid spread across the world—would have been unlikely to have been significantly affected by the provision of crucial information a week or two earlier.^1^ This is because even when such information was available appropriate steps were often not taken, including especially in the United States, from which much further global transmission then occurred. In addition, the resort to recriminations and attempts to attribute blame for the pandemic undermined the chances not just of an effective, coordinated global response to contain the pandemic but also of the establishment of a new era of global cooperation around critical common interests.

It is worth pointing out that for many scientists and clinicians the advent of a novel epidemic, including from coronaviruses, came as no surprise. Indeed, as will be argued below, the multiple ingredients for such an event had existed for years and experts had been warning that it was only a matter of time before the next pandemic emerged (possibly due to a coronavirus, given the history of recent coronavirus incursions from animals to humans). Strenuous attempts had been made both to counter the forces known to be driving the emergence of new coronaviruses from the animal-human interface and to gather the knowledge necessary to expedite the development of diagnostic tests, vaccines, and treatments when they appeared.

In the years preceding the actual emergence of SARS-CoV-2 the scientific work to understand the biology and origins of coronaviruses was generally collaborative (within the limits of everyday scientific competitiveness) and international. It involved laboratories and researchers in China, North America, Singapore, Australia, and elsewhere who generally worked together in a spirit of openness and mutual trust, presenting a remarkable example of the way in which the pursuit of science can serve common values and goals. The joint efforts of these scientists have undoubtedly saved countless lives (Dias 2013; Parrish, et al. 2008; Woolhouse, et al. 2012).

Sadly, much of this achievement has now been destroyed. Driven especially by the policies of the United States and then, regrettably, followed by China itself, the establishment of an effective global response to COVID-19 was blocked by a rapidly assembled politicized response that sought to attribute blame rather than support the continuation of the cooperative framework. Even if the allegations had been true—which, as will be argued below, they were not—the opportunistic use of the disaster in this manner to exacerbate tensions and undermine trust would have been ethically inappropriate, counterproductive, and contrary to the interests of people around the world. It certainly delays the appropriate investigations needed to identify the origins of SARS-CoV-2 (Koopmans, et al. 2021).

## The Laboratory Leak Hypothesis and the Evidence

It is not yet clear exactly where the idea for the story of a lab leak originated but there was never any substantive evidence to support it. Nonetheless, over a short period of promotion by the United States it was actively taken up by the Australian government (ironically, with devastating long-term consequences for the Australian economy and the livelihoods of many Australian farmers), followed by partisan political commentators from a number of other countries.

The central hypothesis, which might better be stated as an allegation, was that SARS-CoV-2 had been deliberately engineered at the Wuhan Institute of Virology (WIV) as a result of unethical “gain of function” research and then, probably inadvertently, released to the public (amplified at the Huanan wet market) by a laboratory worker. From the beginning, the only “evidence” that was advanced in support of this claim was: (1) the proximity of the laboratory to the market; (2) assertions that features of the SARS-CoV-2 genome that contribute to pathogenicity and transmission (for example, the presence of a furin cleavage site, and binding to human ACE2 receptors) must have been inserted deliberately; (3) that these gain of function studies may have been done with malign intent; and (4) allegations that the Chinese authorities refused to provide full access to laboratory records in response to demands for accountability, supposedly indicating the presence of a cover-up.

The first claim is probably spurious. The WIV is located at least 20 kilometres from the Huanan wet market, the agreed epicentre of the Wuhan outbreak. It is an international leader in bat virus research, including with SARS-related coronaviruses (SARSr-CoV), and has published extensively, but no evidence has been presented that SARS-CoV-2 was ever studied at the WIV prior to the emergence of disease at the Huanan wet market in December 2019, nor that there were other unpublished similar viruses isolated in the laboratory. Laboratory leaks can happen, generally when viruses have been isolated in tissue culture where viral loads are very high, rather than directly from clinical samples. The SARSr-CoV sequenced at the WIV, RaTG13 (collected in 2013), is one of the viruses most closely related to SARS-CoV-2 but was only available as a viral sequence and not as a viral isolate. Research and public health laboratories, the latter managed through the Chinese Center for Disease Control (CDC) system are found in provinces and many large cities in China, where research into important and dangerous disease is well supported. One might speculate that wherever an urban outbreak in China might occur it would be likely that a viral laboratory might be found reasonably nearby. In Wuhan, in addition to the research focused WIV, there are Wuhan CDC and Hubei Province CDC public health laboratories as well as university and veterinary laboratories. This is similar to other major cities in China.

The second claim has now been comprehensively disproved in work by scientists from Australia and elsewhere, which have shown that the SARS-CoV-2 features that allow ready human transmission can all arise by natural evolutionary processes in animals (Andersen et al., 2020; Dwyer 2022; Holmes et al. 2021). Through meticulous analysis of the viral sequence these studies have identified no evidence of human intervention, through genetic modification, including addition or deletion of sequences. In addition, they have shown that all relevant features of the virus occur in nature in settings that make a natural evolutionary process both feasible and plausible. Coronavirus genomic similarities in bats (and pangolins), the characteristics of bat SARSr-CoV, the known coronavirus genomic plasticity, the recent historical emergence of other human and animal coronaviruses, and the complete lack of evidence of an actual laboratory leak means that *in vivo* coronavirus recombination or mutation in host and intermediate animals are overwhelmingly likely as the driver of human SARS-CoV- 2 (Andersen, et al. 2020; Holmes, et al. 2021).

The third claim has no evidence or credible basis, and indeed, no attempt has even been made to provide any relevant data to support it. The fourth claim—that Chinese authorities have failed to provide access to all relevant data to interested parties—remains open and would require further evidence to be resolved. However, it is noted that a forensic investigation of the WIV was not within the terms of reference of the WHO Study Group and, despite calls from some parties, there has been no formal process within which such an investigation has been required. Nonetheless, recently published analysis by American and European researchers of metagenomic sequencing data publicly available on GISAID has demonstrated the co-occurrence of SARS-CoV-2 and the genetic material of susceptible wildlife in environmental samples from the Huanan wet market during the start of the COVID-19 pandemic, indicating strongly that the vector at this location was an animal (possibly a racoon dog) rather than a human. These findings mitigate against the hypothesis that the virus was introduced to the market through a human host (Liu et al., 2023a; SAGO 2023).

Perhaps predicably, the raising of unsupported allegations against Chinese scientists in a context considered by the Chinese government to be scurrilous and unfair directly undermined the possibility of a much-needed collaborative investigation into the actual causes of the unfolding disaster. Instead, the Chinese countered, unhelpfully, with unfounded allegations of their own, suggesting that the virus could have been deliberately engineered in the United States and released as an act of sabotage by an American undercover agent during the military games which had been held in Wuhan a few months before the index super-spreader event. It should be noted that the U.S.A National Intelligence Council (NIC) has given an updated assessment, with varying levels of confidence, of the origins of SARS-CoV-2: China did not develop SARS-CoV-2 as a biological weapon, SARS-CoV-2 had not been genetically engineered; Chinese authorities were not aware of SARS-CoV-2 before the initial outbreak; and that that infection was most likely caused by natural exposure to an animal infected with it or a close progenitor (Office of the Director of National Intelligence, 2023).

While the American, Australian, and Chinese claims were all theoretically possible, as mentioned, they have now been discredited as there are no good data to support them, and we have to look elsewhere for the “origins” of the new virus. Luckily, here, the evidence is plentiful. A substantial body of knowledge, supported by a great deal of data, favours the original hypothesis of most informed experts: that the emergence of the SARS-CoV-2 virus occurred, like its predecessors, as a result of the well-documented processes of mutation within animal reservoirs followed by cross-species transmission to humans. This is a process that has occurred many times in the past with coronaviruses and other pathogens and, as noted, was long expected by scientists and other experts in the field (Bloom 2021; Ferreira, et al. 2021; Slingenbergh, et al. 2004).

The continuing, well characterized mutations of the virus support this understanding and underline the perverse nature of the allegations against the Wuhan scientists. While the currently circulating variants are significantly different from the original Wuhan version, incorporating up to a hundred or more novel mutations, it is now universally accepted that this is the result of the remarkable ability of this family of viruses to evolve. Indeed, no one has claimed that the new variants of the virus (such as Alpha, Beta, Delta, Omicron etc.), which variably emerged from the United States, Europe, South Africa, India, and South America, were deliberately engineered in a laboratory. If it is possible to accept such a natural process of mutation now, the likely role of similar processes before the appearance of the “Wuhan strain” became known should be all too apparent.

## From Where Did SARS-CoV-2 Really Come?

How then should we understand the real origin of COVID-19? If it is accepted that the novel virus evolved silently in nature, perhaps over a long period of time and then made the tragic leap to humans, questions remain about why such a leap occurred and whether additional factors were in operation that facilitated the entire process. This is obviously of fundamental importance if we are to take steps to prevent further pandemics occurring.

Fortunately, here too, a great deal of knowledge exists that draws on many decades of work by scientists in a number of fields from around the world. This work has been stimulated by the recognition of an increasing incidence of new zoonotic infections over the last forty years.

While complex and multifactorial, as a result of this research many of the factors underlying the increasing incidence of zoonotic infections are now well recognized. They include demographic changes, increased travel and tourism, changed farming and land use practices, variations in wildlife populations, changing trade patterns, healthcare factors, and the effects of climate change (Bhattacharya, et al. 2020; Cordoba-Aguilar et al., 2021; Gilbert 2022; Pekar, et al. 2022). Of these, a driver of increasing importance has become the environmental disruptions associated with climate change, which have transformed the relationships between animal species and human societies. This process is in itself highly complex, operating through a number of factors, including the destruction of habitats, major changes in food systems, altered migration patterns of wild animals, and the variable and sometimes unpredictable impact of changes in weather patterns (Carlson, Albery, and Phelan 2021; Tollefson 2020; Friend 2006).

In other words, regardless of events that may or may not have occurred in the last three weeks of 2019, the key, underlying “cause” of the COVID-19 pandemic was the long slow process of change in the relationships between humans and animal species, driven by overlapping and interwoven social, economic, and environmental forces linked to the now familiar process of climate change.

In this complex setting, therefore, it is not an exaggeration to regard the advent of novel zoonotic infections as one of the many consequences of climate change, alongside the more widely recognized floods, droughts, bushfires, and other extreme weather events. It is also possible to infer that of all the effects of climate change the increasing frequency of pandemics similar to COVID-19 may ultimately prove to be the most destructive.

## The Story of Origins As We Now Understand It

So here is the most likely sequence of events. Somewhere in southern China or a nearby country a coronavirus present in microbats found its way into one or more other animal hosts: the intermediate host remains elusive, but a large range of animals can be infected with SARS-CoV-2, including pangolins and felines. Prior to late 2019, perhaps, the infected bats or intermediate animals had had little contact with humans or the virus could not infect or cause noticeable human disease.

Through the normal processes of human movement and intercourse, the new virus was quickly passed on to other people or animals and found its way to Hubei province. An infected animal was brought to the market for sale, resulting in initial transmission to twenty-one people. Over the next two weeks, enough people became sick with severe pneumonia to alert doctors and public health officials, leading to the announcement warning the world of the dangerous new disease. The market was closed the following day and vigorous efforts were made to identify and isolate contacts.

Three weeks later it was clear that these measures could not contain the epidemic and the Chinese authorities took the brave and unprecedented step of locking down the entire city. Although exact data about the number of infections remain uncertain, it is clear that this and related actions proved effective in controlling the spread of the virus in China.

However, it was already too late to stop the spread internationally. As a result of the extent and rapidity of international travel, tourism, and trade, within a few weeks it was present in Taiwan, South Korea, many European countries, the United States, and Australia.

This much is known. What we now have to find out is what had happened in the months or years leading up to December 2019 and whether, in retrospect, anything could have been done to prevent the impending disaster. To answer these questions we need to unpick the multiple steps in the fatal chain of transmission from a harmless wild animal virus to a deadly human infection.

It is critically important that we understand the evolution of this virus because, as with all zoonoses, it will have occurred as a result of both random biological events and responses to environmental pressures. The virus had to acquire mutations allowing human transmission, the original wild animal (for example, a bat) had to be exposed to other species, and the virus had to spread within those species and possibly undergo further mutations. The animal had to come into close contact with a human being who, at the right moment, had to contract the new infection.

Despite the low probability of each individual step, over recent decades an increasing array of viruses has negotiated this entire pathway, including HIV, SARS, MERS, Ebola, Nipah, Lassa, Zika, Hendra, Mpox, influenza, and now SARS-CoV-2. We also know that other seasonal human coronaviruses associated with the common cold (HKU1, 229E, OC43, NL63) have animal origins. This supports the argument that novel factors are operating that increase the chances of exposure, adaptation, infection and spread.

It is likely that these factors are the ones already discussed, including population growth, agricultural expansion, the loss of habitats of wild animals, the loss of traditional food sources, and changing relationships between animal species and between animals and humans. Deforestation and climate change further exacerbate this process, as does increased movement of human populations, through domestic and international travel. The international illegal wildlife trade, inappropriate use of drugs and insecticides and reluctance of governments (or agencies within government) to work together make matters even worse. As previously mentioned, a common feature of these processes, which is likely to exacerbate their impact, if not always to have provided the underlying originating force, is the abiding presence of climate change.

## How Did Science Become so Politicized?

Let’s go back to the beginning. If there was never any credible evidence in support of the laboratory leak hypothesis and the forces driving the appearance of new zoonotic infections are so well documented, how did it occur that public discussion—at least in western countries—became so distracted by the false narrative that the Chinese were to blame for the pandemic?

There is an obvious possible answer to this question: the lab leak theory was a convenient, if cynical, weapon that was devised simultaneously by certain western countries to serve short-term political objectives and to shift the focus away from both their own negligent responses to the pandemic and the urgent need to address the problem of climate change (Egan 2020). While elements of this explanation may well be accurate it still leaves unanswered the question of how it was possible to manipulate the scientific and media discourses in this manner. After all, if science is no more than a presentation of true, objective facts how could it be so readily mobilized for such ignoble purposes?

This remains a subject for ongoing debate among scientists, ethicists, political philosophers, and others. However, a few broad hypotheses may be stated on the basis of well-established, historical experience. Science is, and has always been, “political” in several senses (Funtowicz and Ravetz 2020; Komesaroff 2008; Latour 1988; Ravetz 1971). First, scientific theories have always reflected fundamental philosophical assumptions of an epistemological and ontological nature, as a result of which theories of science can never be extricated from their embeddedness in the prevailing culture. Second, scientists are social actors entrapped by prevailing ideologies, on the basis of which they shape the goals and purposes for which they understand themselves to be striving. Third, the insights of science and the technological developments it makes possible can be applied in many different ways, for both progressive and destructive purposes, embracing both the enhancement of human well-being and the refinement of the instruments of war and destruction.

In other words, science and scientists are not inherently committed to any single set of values, and certainly not one that is directed toward beneficent consequences. This is the work of ethical discourses which may run courses independent of and, at times directly contrary to, those of science itself. There is no substitute for a dynamic process of critical ethical oversight which scrutinizes and, where necessary, redirects the putative ends of those immersed in the scientific enterprise.

Indeed, even the supposed commitment of science to a regime of pure truth is not unambiguous. After all, as the experience of COVID-19 has shown, alleged “facts” are always subject to interpretation, which depends on judgments inextricably linked to the social, political, and philosophical contexts within which the factual propositions are formulated. Further, in the biological sciences especially, certainty is rarely, if ever, attained. Whereas a politically derived assertion can be stated in hyperbolic and peremptory terms, the scientific and public health response can sometimes do no more than encourage circumspection and doubt. The mobilization of social media as an additional source of untestable claims has undermined even further the ability of scientists committed to a disinterested assessment of the evidence. Social media also has allowed personal attack or invective to cloud discussion. Such issues are not limited to investigation of the origins of SARS-CoV-2 but also include responses around mask use, other social distancing methods and vaccination. The usual methods of scientific discourse through peer-reviewed publications and conference presentations are perhaps being overcome by new information pathways in potentially manipulated social and more traditional media. Even the new movement to scientific “pre-print” publication causes complications where non-peer reviewed or revised data are accepted as “reliable” and “accurate.”

Of course, this is not the full story and the breath-taking achievements of science in relation to the pandemic must not be minimized, including the rapid clarification of the nature and origin of the causative agent, the establishment of effective public health strategies to limit transmission and protect vulnerable populations, and the development of vaccines and antiviral therapies. These achievements have produced incalculable benefits for people across the world. Nonetheless, contemporaneous attempts to exploit these very achievements in the service of sectional interests linked to global power struggles (including within international organizations) poses the risk of compromising whatever remains of the ethos of science at its core.

## Conclusions

The story of the response to the COVID-19 pandemic has been bittersweet but may still offer some lessons (Cordoba-Aguilar, et al. 2021; Keuscha, et al. 2022; McNeely 2021; Sachs, et al. 2022; Sanchez, et al. 2022).

On the one hand, we now know a lot about SARS-CoV-2 including many of the factors that gave rise to it and how to limit its harmful effects. We understand the link between novel zoonotic infections and the inexorable forces of climate change, and what actions are needed to avoid future pandemics. On the other hand, we have learnt that neither science nor scientists are “pure” and that all knowledge is vulnerable to appropriation by regimes of power committed to contrary goals.

Some clear options are available. These could include: the development of a coordinated system for identifying potentially dangerous pathogens to track naturally occurring mutations and map interactions between species where the risk of transition may arise; the preservation of native habitats, prevention of deforestation, and reduction of pressures on wild animals to enter human spaces in search of food; precise targeting of infection control procedures such as health monitoring and quarantine, vector control, and culling of reservoir animals; cooperative efforts to develop diagnostic tests, new drugs and vaccines; and the establishment of early warning systems to ensure rapid international action when new infections do arise (Keuscha, et al. 2022). It is important that such processes are not limited to those countries that can afford such systems but are applied evenly. Or they could support the further undermining of the institutions of science and knowledge; the accelerated erasure of the systems of cooperation and trust that have been constructed so painstakingly over decades; and the deployment of short-term recriminations, ideological rivalries, and political ambitions in place of our responsibilities to the planet and to each other.

The COVID-19 experience may be interpreted as sounding an urgent call for a renewal of ethical discourses about the ends of science and of humanity. We can only hope that this call will be heeded.

## Footnotes

1 Arguably, among the benefits of faster release of the early sequence data may have slightly expedited development of diagnostic tests.
